# Supplementary material for: Negatively charged nanoporous membrane for a dendrite-free alkaline zinc-based flow battery with long cycle life
Source: Nat Commun. 2018 Sep 13;9:3731. doi: 10.1038/s41467-018-06209-x (PMC6137156; doi:10.1038/s41467-018-06209-x)
Supplement: Supplementary file 1 — Supplementary Information [file 41467_2018_6209_MOESM1_ESM.pdf]

Supplementary Information For

Negatively charged nanoporous membrane for a dendrite-free alkaline zinc-based flow battery with long cycle life

by Yuan *et al.*

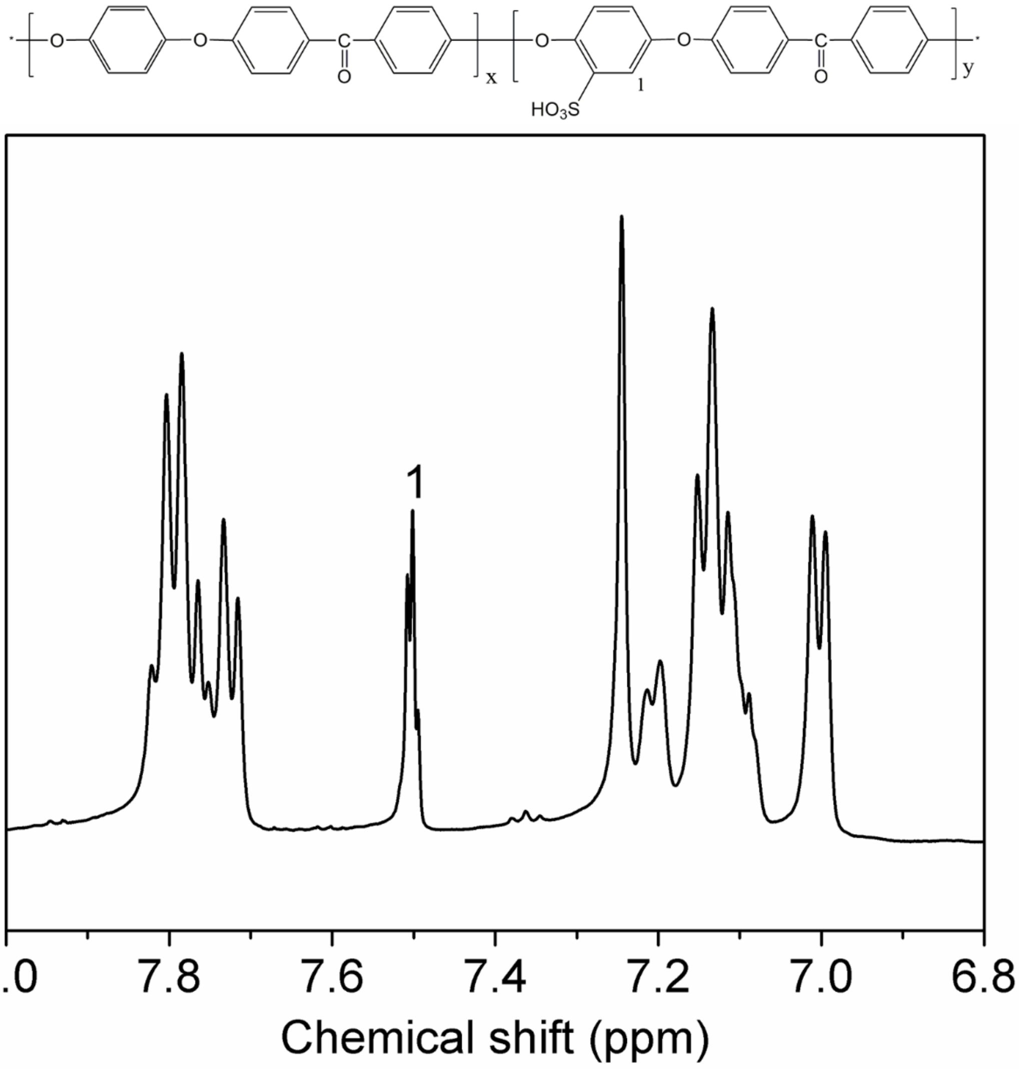

Supplementary Figure 1. Chemical structure and ^{1}H NMR spectrum of sulfonated poly (ether ether ketone) (SPEEK).

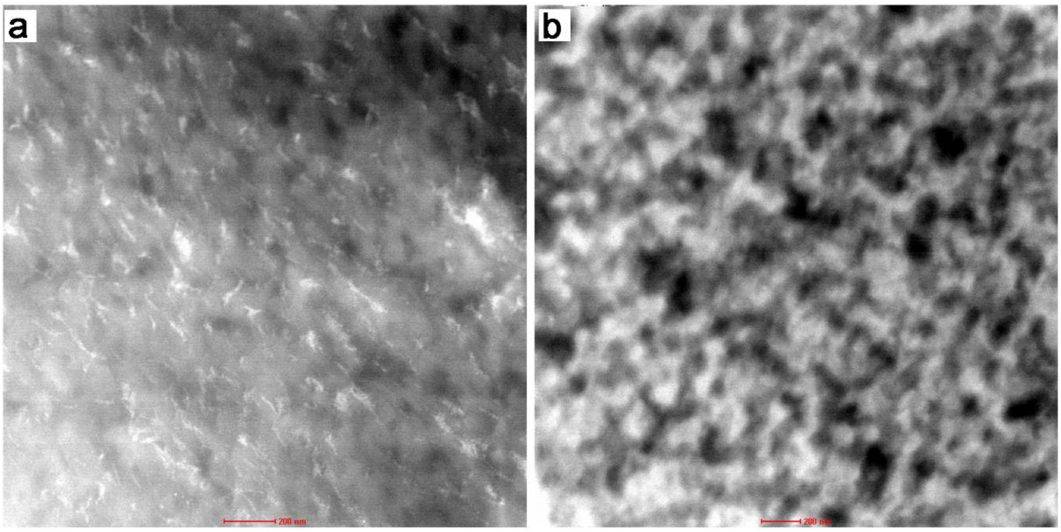

Supplementary Figure 2. STEM image of P20 and P0 membranes. (a) STEM image of P20. **(b)** STEM image of P0. STEM was performed on P20 membrane after Ag^{+} dyeing to investigate the scattering of the negatively charged sulfonated acid groups on the pore walls. For compare, STEM image of P0 membrane stained with $[PdCl_4]^{2-}$ was investigated as well. Both P20 and P0 demonstrated a sponge-like, porous structure (black zones represent the pores). And Ag nanoparticles were distinctly inspected around the pore walls, because of the interaction between the negatively charged sulfonated acid groups and the positively charged Ag^{+}. By contrast, no clearly nanoparticles could be found for the P0 membrane, suggesting that the there is no charged groups in the membrane. Scale bar: 200 nm.

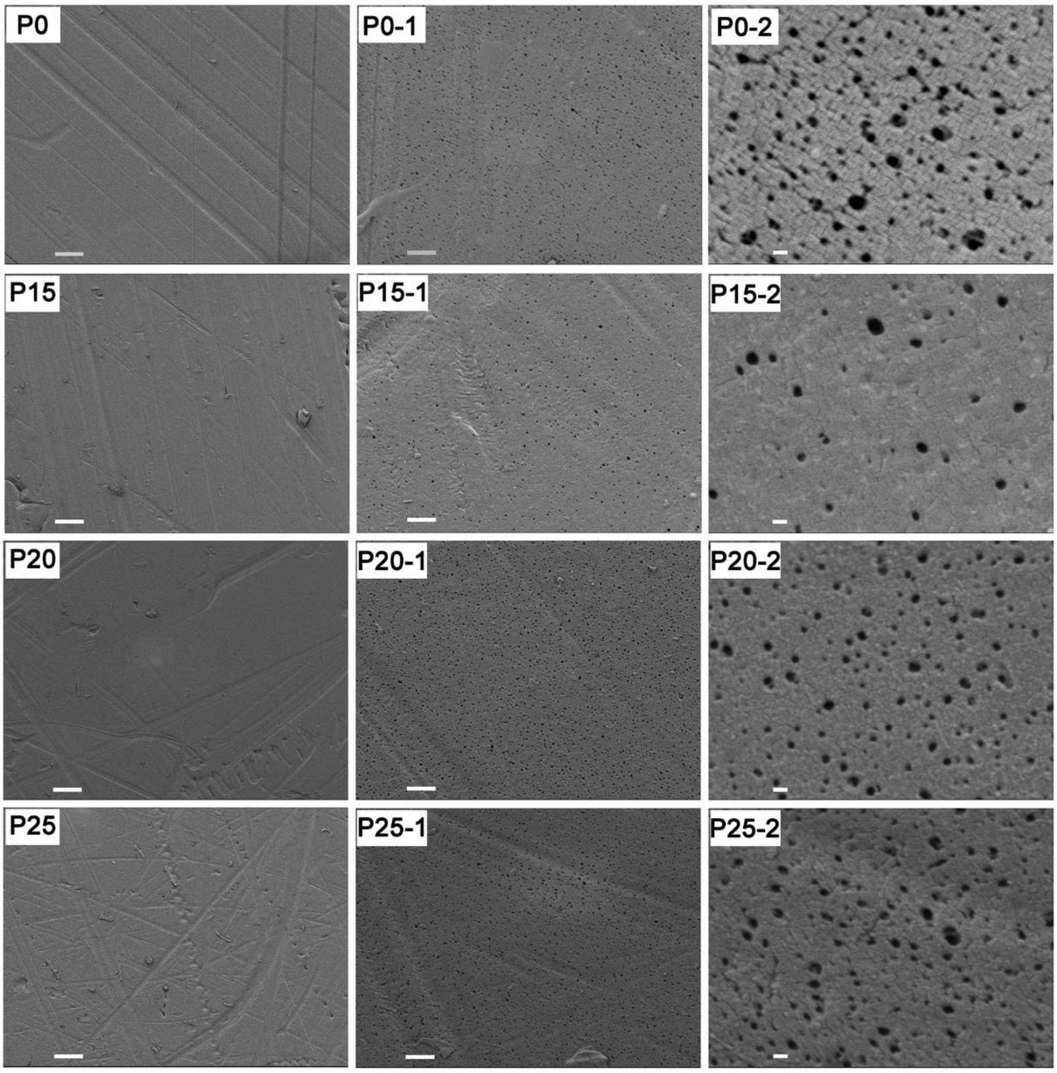

Supplementary Figure 3. Surface morphology. Surface morphologies of negatively charged ordered nanoporous PES/SPEEK membranes and uncharged PES membrane at different magnifications. PX, X represents the SPEEK content in the polymer (for instance, P20, the SPEEK content in the polymer was 20 wt.%). PX-1 and PX-2 are the different magnifications of PX. The scale bar of PX, PX-1 and PX-2 are 10 μm, 1 μm and 100 nm, respectively.

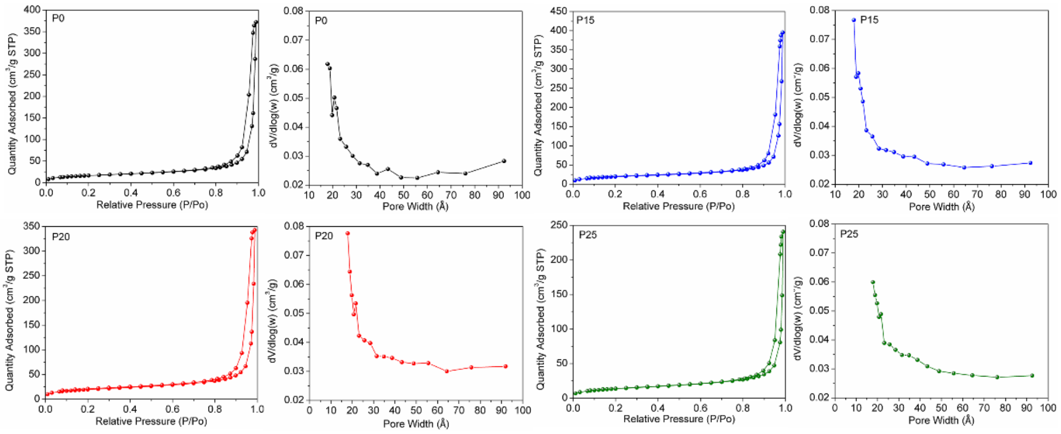

Supplementary Figure 4. Pore size distribution. N_2 sorption isotherms and pore size distribution curve of the prepared nanoporous membranes (determined by Barrett-Joyner-Halenda (BJH) analysis).

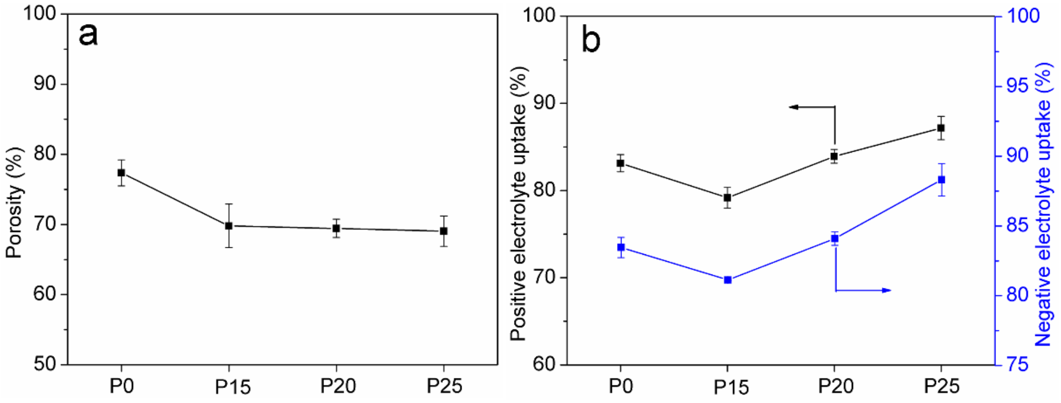

Supplementary Figure 5. Physics property of the prepared membranes. (a) Porosity and **(b)** electrolyte uptake of the prepared nanoporous membranes. Values are mean +/– s.d. of two independent experiments.

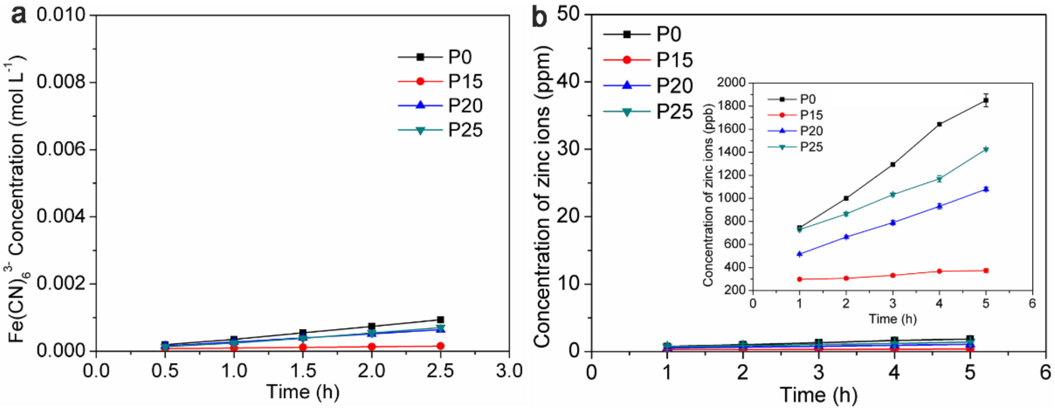

Supplementary Figure 6. The permeability of ferricyanide and zincate ions. The permeability of **(a)** $Fe(CN)_6^{3-}$ and **(b)** $Zn(OH)_4^{2-}$ ions through the prepared nanoporous membranes. Values are mean +/– s.d. of two independent experiments.

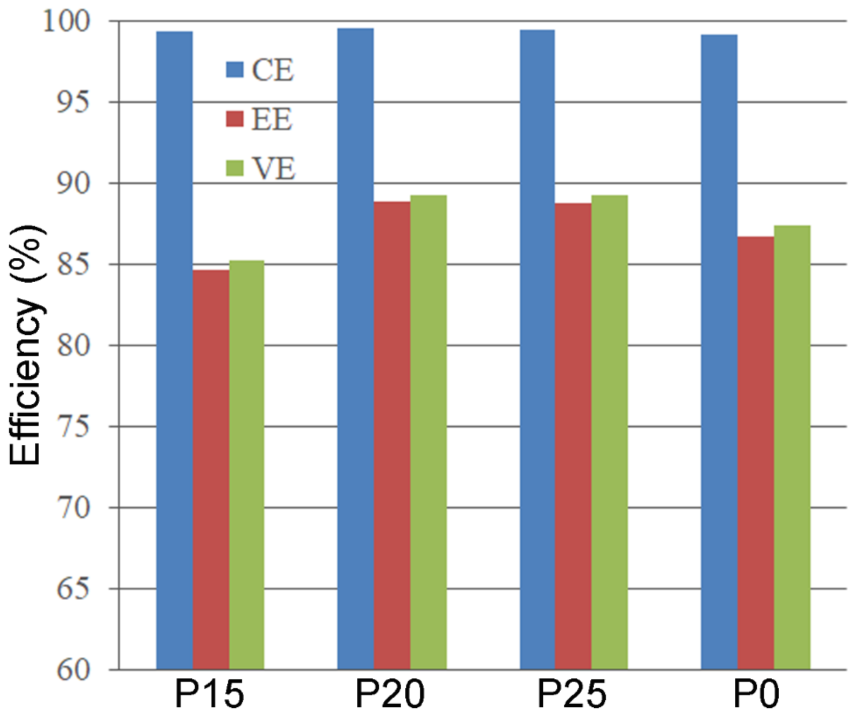

Supplementary Figure 7. Battery performance. The efficiencies of alkaline zinc iron flow battery with P15, P20, P25 and P0 membranes at the current density of 80 mA cm^{-2}.

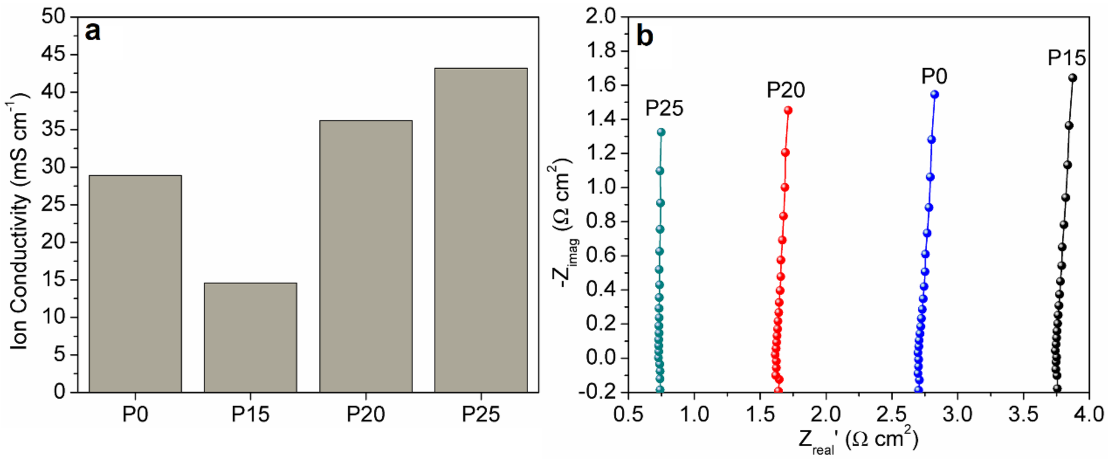

Supplementary Figure 8. Ion conductivity and EIS measurement. (a) Ion conductivity and **(b)** EIS measurement of the prepared nanoporous membranes.

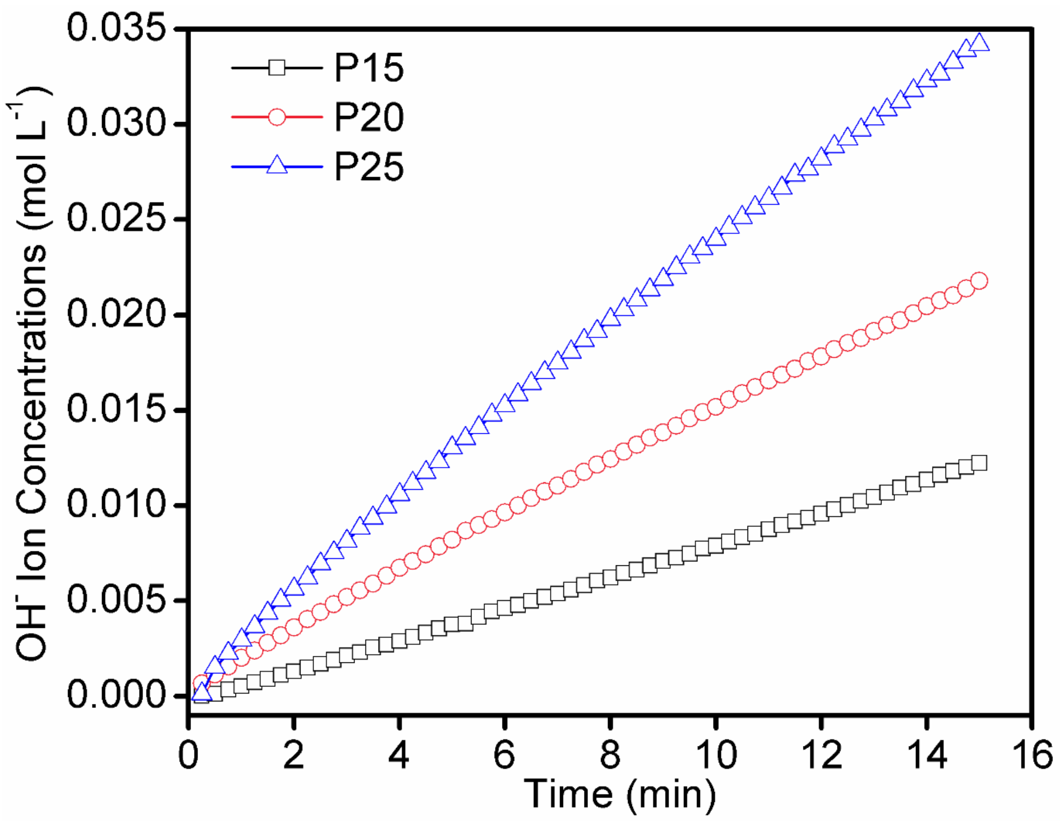

Supplementary Figure 9. OH^- permeability. OH^- concentration versus time at the diffusion side of a diffusion cell using P15, P20 and P25 membranes. Note that the permeation rate of OH^- also stands for the permeability of Na^+ by side since a positively charged ion must pass through the membrane to balance the negatively charged OH^-.

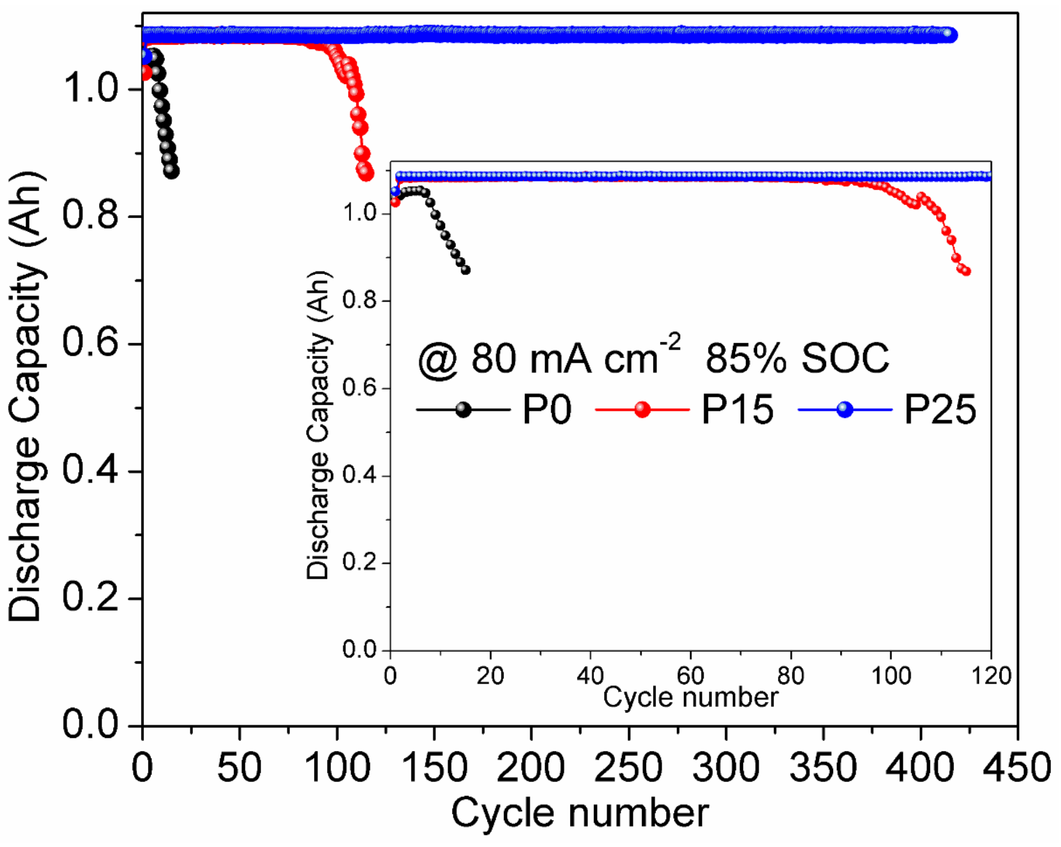

Supplementary Figure 10. Cycling performance. Accelerated cycling experiment of the alkaline zinc iron flow battery with SOC of 85% using P0, P15 and P25 membranes at the current density of 80 mA cm^{-2}.

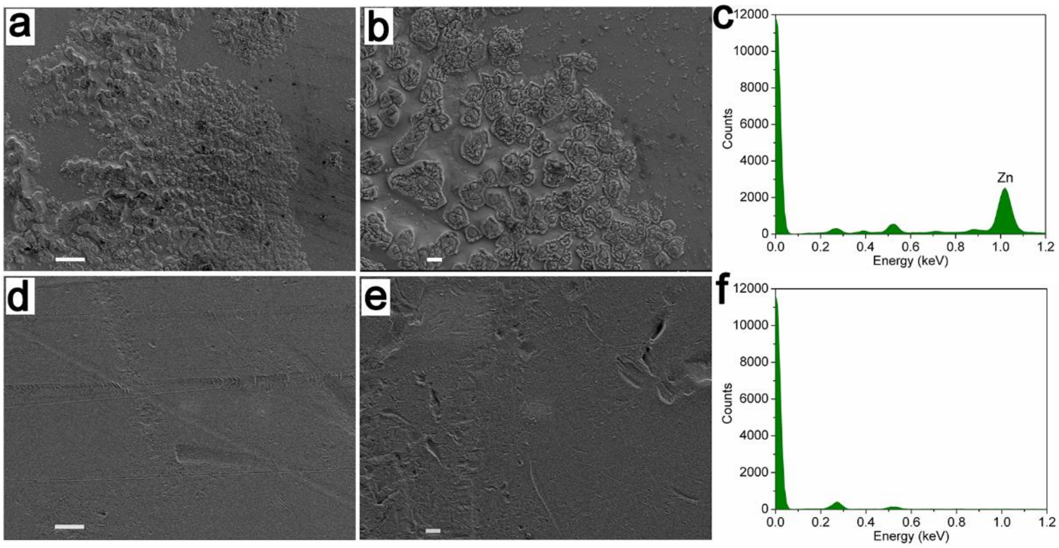

Supplementary Figure 11. The surface morphology of P0 and P20 membranes at the end of battery charging. (a) The surface morphology of P0 membrane at the end of 54th charge. **(b)** Magnified surface morphology of P0 in panel a. **(c)** Corresponding EDS spectrums of P0 membrane in panel b, where obvious zinc metal (dendrites) was pierced into P0 membrane. **(d)** The surface morphology of P20 membrane at the end of 183th charge. **(e)** Magnified surface morphology of P20 in panel d. **(f)** Corresponding EDS spectrums of P20 membrane in panel e, where no zinc element can be detected at the end of charge. Scale bars of a and d are 10 μm. Scale bars of b and e are 1 μm.

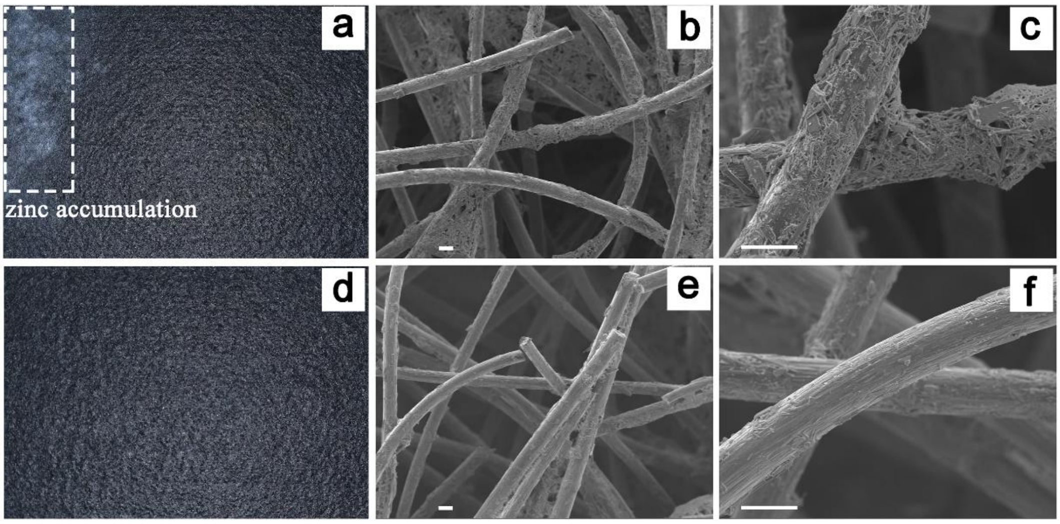

Supplementary Figure 12. The morphology of carbon felt electrode of the battery using P0 and P20 membranes. (a) Optical image of the negative electrode at the end of 65th discharge for the alkaline zinc-iron flow battery assembled with a P0 membrane. **(b)** SEM image of zinc accumulation in the carbon felt in panel a. **(c)** Magnified SEM image of zinc accumulation in panel b. **(d)** Optical image of the negative electrode at the end of 186th discharge for the alkaline zinc-iron flow battery assembled with a P20 membrane. **(e)** SEM image of carbon felt in panel a. **(f)** Magnified SEM image of carbon felt in panel e, no obvious zinc accumulation can be found for the battery using a P20 membrane. Scale bar, 10 μm.

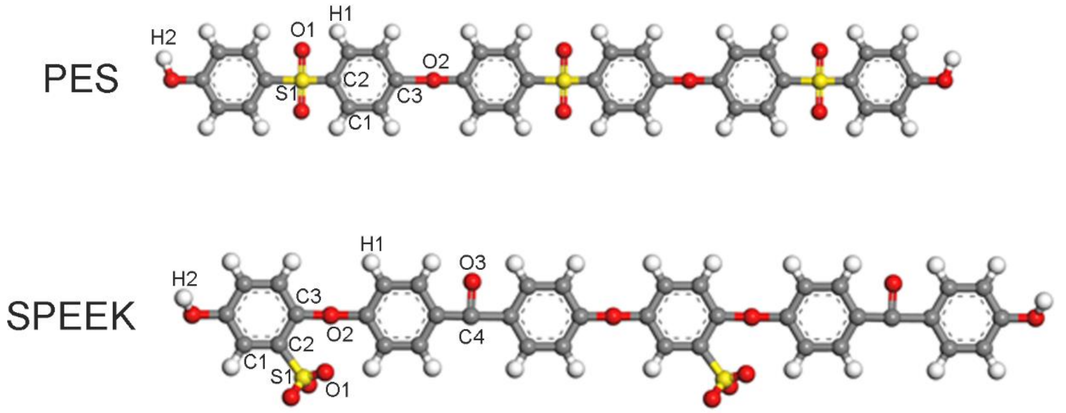

Supplementary Figure 13. Fragmented cluster models of PES and SPEEK, respectively.

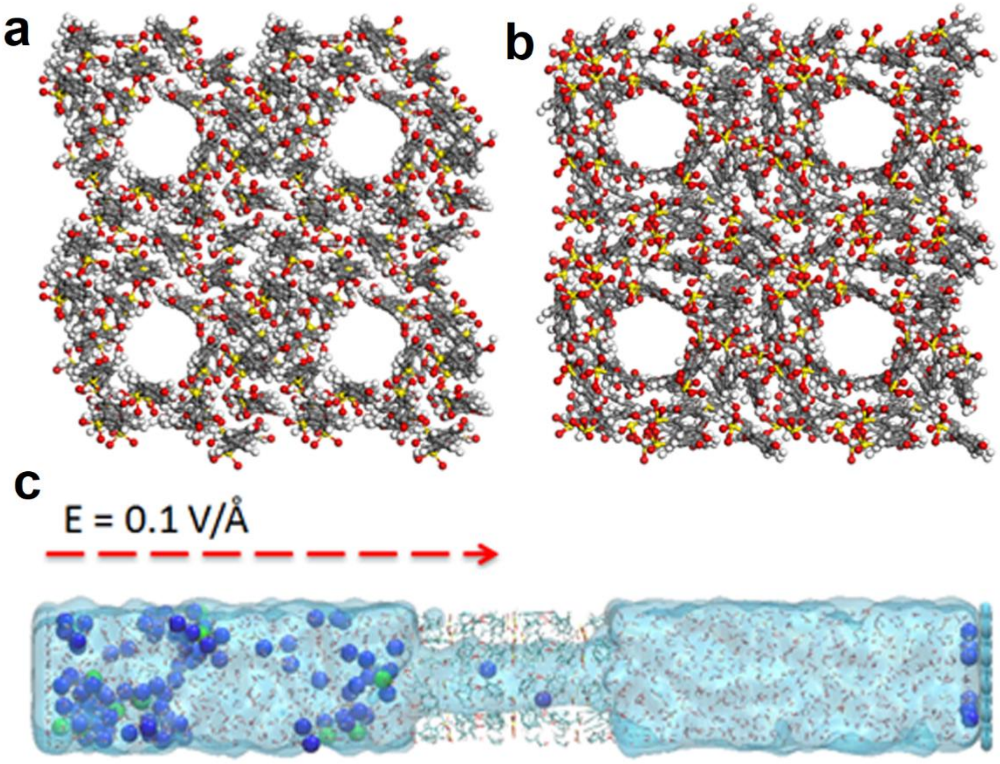

Supplementary Figure 14. Simulation results. Ball-and-stick models of **(a)** P0 and **(b)** P20, respectively. **(c)** The studied model system.

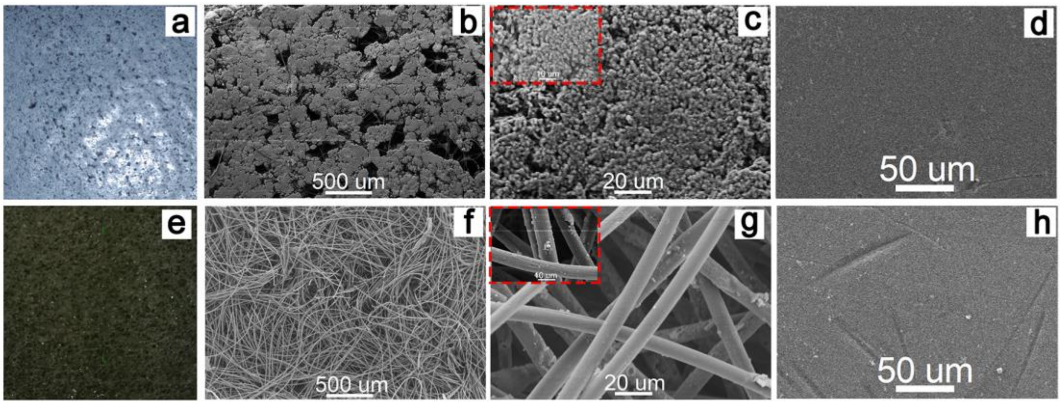

Supplementary Figure 15. The morphology of the carbon felt electrode and P20 membrane at the end of charge and discharge. **(a)** Optical image of the negative electrode at the end of charge for the alkaline zinc-iron flow battery assembled with a P0 membrane (27 cycles at 40 mA cm^{-2} with nearly 8 or 7 h for each plating/stripping step). **(b)** SEM image of zinc metal in the carbon felt in panel a. **(c)** Magnified SEM image of zinc metal in panel b. **(d)** The corresponding surface morphology of P20 membrane at the end of charge. **(e)** Optical image of the negative electrode at the end of discharge for the alkaline zinc-iron flow battery assembled with a P0 membrane (27 cycles at 40 mA cm^{-2} with nearly 8 or 7 h for each plating/stripping step). **(f)** SEM image of zinc metal in the carbon felt in panel e. **(g)** Magnified SEM image of zinc metal in panel f. **(h)** The corresponding surface morphology of P20 membrane at the end of discharge.

Supplementary Table 1. Partial charge of the cluster models of SPEEK and PES using CHELPG charge.

SPEEK		PES	
Element	Charge	Element	Charge
S1	0.986	S1	0.806
O1	-0.598	O1	-0.481
O2	-0.384	O2	-0.424
O3	-0.532	C1	-0.135
C1	-0.154	C2	-0.094
C2	-0.045	C3	0.404
C3	0.341	H1	0.115
C4	0.43	H2	0.392
H1	0.108		
H2	0.365		
