# Supplementary material for: Negatively charged nanoporous membrane for a dendrite-free alkaline zinc-based flow battery with long cycle life
Source: Nat Commun. 2018 Sep 13;9:3731. doi: 10.1038/s41467-018-06209-x (PMC6137156; doi:10.1038/s41467-018-06209-x)
Supplement: Supplementary file 3 — Description of Additional Supplementary Files [file 41467_2018_6209_MOESM3_ESM.pdf]

Scientific Description of Supplementary Movies

Supplementary Movie 1 (P0): Molecular dynamics simulation result of the rejection between the negatively charged $Zn(OH)_4^{2-}$ ions and the uncharged P0 nanoporous membrane as well as the Na^+ ions transportation through the membrane.

Supplementary Movie 2 (P20): Molecular dynamics simulation result of the rejection between the negatively charged $Zn(OH)_4^{2-}$ ions and the negatively charged P20 nanoporous membrane as well as the Na^+ ions transportation through the membrane.
